# Impact of phosphorylation of heat shock protein 27 on the expression profile of periodontal ligament fibroblasts during mechanical strain

**DOI:** 10.1007/s00056-022-00391-w

**Published:** 2022-04-21

**Authors:** Agnes Schröder, Kathrin Wagner, Fabian Cieplik, Gerrit Spanier, Peter Proff, Christian Kirschneck

**Affiliations:** 1 Department of Orthodontics, University Hospital Regensburg, Franz-Josef-Strauss-Allee 11, 93053 Regensburg, Germany; 2 Department of Operative Dentistry and Periodontology, University Hospital Regensburg, Franz-Josef-Strauss-Allee 11, 93053 Regensburg, Germany; 3 Department of Cranio-Maxillo-Facial Surgery, University Hospital Regensburg, Franz-Josef-Strauss-Allee 11, 93053 Regensburg, Germany

**Keywords:** Heat shock proteins, Orthodontic tooth movement, Compressive forces, Bone remodeling, Osteogenesis, Hitzeschockproteine, Kieferorthopädische Zahnbewegung, Druckkräfte, Knochenremodelling, Osteogenese

## Abstract

**Purpose:**

Orthodontic tooth movement is a complex process involving the remodeling of extracellular matrix and bone as well as inflammatory processes. During orthodontic treatment, sterile inflammation and mechanical loading favor the production of receptor activator of NF-κB ligand (RANKL). Simultaneously, expression of osteoprotegerin (OPG) is inhibited. This stimulates bone resorption on the pressure side. Recently, heat shock protein 27 (HSP27) was shown to be expressed in the periodontal ligament after force application and to interfere with inflammatory processes.

**Methods:**

We investigated the effects of phosphorylated HSP27 on collagen synthesis (*COL1A2 *mRNA), inflammation (*IL1B* mRNA, *IL6* mRNA, PTGS2 protein) and bone remodeling (RANKL protein, OPG protein) in human periodontal ligament fibroblasts (PDLF) without and with transfection of a plasmid mimicking permanent phosphorylation of HSP27 using real-time quantitative polymerase chain reaction (RT-qPCR), western blot and enzyme-linked immunosorbent assays (ELISAs). Furthermore, we investigated PDLF-induced osteoclastogenesis after compressive strain in a co-culture model with human macrophages.

**Results:**

In particular, phosphorylated HSP27 increased gene expression of *COL1A2* and protein expression of PTGS2, while *IL6* mRNA levels were reduced. Furthermore, we observed an increasing effect on the RANKL/OPG ratio and osteoclastogenesis mediated by PDLF.

**Conclusion:**

Phosphorylation of HSP27 may therefore be involved in the regulation of orthodontic tooth movement by impairment of the sterile inflammation response and osteoclastogenesis.

**Supplementary Information:**

The online version of this article (10.1007/s00056-022-00391-w) contains supplementary material, which is available to authorized users.

## Abstract (de)

**Hintergrund:**

Die kieferorthopädische Zahnbewegung ist ein komplexer Prozess, der den Umbau der extrazellulären Matrix und des Knochens sowie entzündliche Prozesse umfasst. Während der kieferorthopädischen Behandlung begünstigen sterile Entzündungen und mechanische Belastungen die Produktion von RANKL („receptor activator of NF-κB ligand“). Gleichzeitig wird die Expression von OPG (Osteoprotegerin) gehemmt. Dies stimuliert die Knochenresorption auf der Druckseite. Kürzlich wurde gezeigt, dass das Hitzeschockprotein 27 (HSP27) im parodontalen Ligament nach Krafteinwirkung exprimiert wird und in die Entzündungsprozesse eingreift.

**Methoden:**

Wir untersuchten die Auswirkungen von phosphoryliertem HSP27 auf die Kollagensynthese (*COL1A2* mRNA), die Entzündung (*IL1B* mRNA, *IL6* mRNA, PTGS2-Protein) und den Knochenumbau (RANKL-Protein, OPG-Protein) in humanen parodontalen Ligamentfibroblasten (PDLF) mittels RT-qPCR („real-time quantitative polymerase chain reaction“) Western Blot und ELISAs („enzyme-linked immunosorbent assays“), ohne und mit Transfektion eines Plasmids, das die permanente Phosphorylierung von HSP27 nachahmt. Darüber hinaus untersuchten wir die durch PDLF induzierte Osteoklastogenese nach Druckbelastung in einem Kokulturmodell mit humanen Makrophagen.

**Ergebnisse:**

Das phosphorylierte HSP27 erhöhte die Genexpression von *COL1A2* und die Proteinexpression von PTGS2, während die *IL6*-mRNA-Spiegel reduziert wurden. Außerdem beobachteten wir eine steigernde Wirkung auf das RANKL/OPG-Verhältnis und die durch PDLF vermittelte Osteoklastogenese.

**Schlussfolgerung:**

Die Phosphorylierung von HSP27 könnte daher an der Regulierung der kieferorthopädischen Zahnbewegung durch Beeinträchtigung der sterilen Entzündungsreaktion und der Osteoklastogenese beteiligt sein.

## Introduction

Numerous cell types and signal substances play a role in orthodontic tooth movement. Based on the force exerted, tensile and compressive zones can be distinguished from each other in the periodontal ligament. At tensile zones, there is increased bone formation, whereas alveolar bone is resorbed at compression zones [22]. The cells use different mechanisms to detect mechanical strain. Both the cytoskeleton [25] and membrane-bound molecules such as integrins [30, 48], connexins [16, 44] and ion channels [17, 32] can be activated by pressure, triggering downstream effects. This includes the synthesis of two key proteins of bone resorption, receptor activator of NF-kB ligand (RANKL) and osteoprotegerin (OPG) [38]. Osteoclast-dependent bone resorption is the limiting factor of tooth movement [10]. During orthodontic tooth movement, RANKL is produced by periodontal ligament fibroblasts (PDLF), mesenchymal stem cells, lymphocytes, osteoblasts, and osteocytes and promotes the differentiation of osteoclasts from osteoclast progenitor cells by binding to the RANK receptor on osteoclast progenitor cells [14, 33, 45–47]. OPG is a RANKL decoy receptor, as OPG prevents binding of RANKL to RANK [38]. Accompanying tooth movement, a sterile inflammation takes place in the periodontal ligament [10]. Pressure loading induces the synthesis and release of pro-inflammatory cytokines such as interleukin-1β (IL-1B) and IL6 in the early phase of tooth movement [14, 26, 39]. Thus, bone remodeling and a sterile inflammatory response characterize orthodontic tooth movement.

Among others, heat shock proteins (HSPs) can influence these processes. Several HSPs have already been linked with orthodontic tooth movement [3, 23, 24, 37]. During bone formation at tension areas, HSPs act as molecular chaperones, which assist the maturation of bone morphogenetic proteins [24]. HSPs are involved in several processes, including protein transport, protein folding, and the assembly and disassembly of protein structures [50].

They support protein refolding and become active in misfolding, where they either refold the misfolded proteins or release them for degradation [50]. HSPs also play a significant role in immunomodulatory signaling pathways in response to stress and are therefore also considered important biomarkers [50]. Some HSPs are involved in the production of pro-inflammatory cytokines, such as IL1ß and IL6. HSPs are classified according to their molecular size. This results in seven families: HSP110, HSP100, HSP90, HSP70, HSP60, HSP40 and small HSPs, which have an approximate size of 15–30 kDa. HSP27 thus belongs to the small HSPs. Wolf et al. found that HSP70 in PDLF has an effect not only on osteoclast differentiation, but also on proliferation, wound healing, and apoptosis [42]. Recently, HSP27 was shown to inhibit the inflammatory response in intestinal epithelial cells via the NF-κB signaling pathway [49]. HSP27 is associated with protein folding, cell migration, cell metabolism, cell differentiation, cell growth, signal transduction and apoptosis [15]. HSP27 is expressed by most cells, although the amount expressed varies greatly depending on the cell type. Synthesis of HSP27 is induced by heat, stress, estrogens, and nerve injury [15]. Posttranslational modifications can strongly influence the functions of HSP27. It can act as a chaperone, stabilizing denatured or aggregated proteins and folding them back to their original form [12, 19]. Phosphorylation, which is triggered by oxidative stress [28], abolishes oligomerization, resulting in increased chaperone activity of monomers [2, 11]. The localization of HSP27 is not limited to the cytosol, but has also been detected in the nucleus [1]. In addition, HSP27 also has an anti-apoptotic effect [4, 5]. This has a promoting effect on cancer progression and metastasis. In this regard, HSP27 overexpression not only leads to increased invasiveness, but also to resistance to chemotherapeutic treatments [8, 27, 29]. Despite years of research and numerous findings, the functions of HSP27 have not been fully elucidated.

Increased expression and phosphorylation of HSP27 was detected in the periodontal ligament already 10 min after force application in a mouse model [23, 37]. Therefore, our aim was to verify whether HSP27 exhibits an anti-inflammatory effect also in PDLF and thus could be a potential target to influence orthodontic treatments. Furthermore, the functional diversity of heat shock proteins suggests other previously unknown roles in cell homeostasis. Therefore, a possible influence on collagen synthesis as well as mediators of bone formation and degradation was also investigated.

## Materials and methods

### Cultivation of human periodontal ligament fibroblasts

Primary human periodontal ligament fibroblasts (PDLF) were isolated from periodontal tissue remnants of extracted caries-free teeth, which were extracted for medical reasons, and grown in complete medium RPMI1640 (61870-010, Gibco™, Carlsbad, CA, USA) with 10% bovine calf serum (FBS; P30-3302; PAN-Biotech, Aidenbach, Germany), 1% L‑glutamine (SH30034. 01, GE Healthcare, Chicago, IL, USA), 100 µM ascorbic acid (A8960, Sigma-Aldrich, St. Louis, MO, USA), and 1% antibiotics/antimycotic (A5955, Sigma-Aldrich, St. Louis, MO, USA) under cell culture conditions. The fifth to seventh passage of a pool of PDLF from six different patients (3 females; 3 males; age: 17–27 years), which had been previously characterized [31], were used for experiments.

#### Kinetic experiments

To evaluate under which conditions stable phosphorylation of HSP27 could be detected, kinetic experiments were performed by loading PDLF with variable magnitudes of compressive forces using plates of zirconium oxide with different heights resulting in different weights per cm^2^ (2 g/cm^2^, 4 g/cm^2^, 6 g/cm^2^; supplemental Fig. 1a) for various time spans (1 h, 2 h, 4 h, 6 h, 24 h, 48 h).

#### Transfection experiments

The plasmid pHSP27^+^ (MS16‑6, MWG eurofins, Ebersberg, Germany) contains human HSP27 gene with three times aspartate instead of serine. Due to the negative charge of aspartate, permanent phosphorylation occurs. An empty plasmid served as a control plasmid. PDLF were transfected with the corresponding plasmids. For this purpose, 50,000 cells were initially seeded per well of a 24-well plate (662 160, Greiner Bio-One, Frickenhausen, Germany). On the next day, 0.5 μg plasmid DNA (either control or pHSP27^+^) was mixed with 100 μl serum-free RPMI medium, and 0.5 μl TurboFect™ (R0531, Thermo Fisher Scientific, Waltham, MA, USA) and incubated for 20 min at room temperature. Subsequently, transfection of the PDLF was performed by adding 100 μl of the respective transfection mixture to the cells. After an incubation period of another 24 h, the transfected cells were exposed to 6 g/cm^2^ for 6 h (supplemental Fig. 1b). Cell number and lactate dehydrogenase (LDH) release were determined and RNA and protein were isolated for further analysis.

#### Coculture experiments

To investigate the effect of transfection on osteoclast differentiation, a coculture of PDLF and human macrophages (THP‑1; TIB-202; ATCC; Manassas, VA, USA) was established. THP‑1 monocytes were cultured in RPMI (61870-010, Gibco™, Carlsbad, CA, USA) supplemented with 20% FBS (P30-3302; PAN-Biontech, Aidenbach, Germany) and 1% antibiotics/antimycotic (A5955, Sigma-Aldrich, St. Louis, MO, USA). First, suspension THP‑1 monocytes were differentiated to adherent macrophages by addition of 25 ng/ml phorbol 12-myristate 13-acetate (PMA; 19-144, Sigma-Aldrich, St. Louis, MO, USA) for 3 days. PDLFs were seeded in 24-well plates (662 160; Greiner Bio-One, Frickenhausen, Germany) according to the experimental setup for transfection and compressed with 6 g/cm^2^ for 6 h (supplemental Fig. 1c). After the compression period, we checked, whether the suspension THP‑1 monocytes were differentiated into adherent macrophages under the microscope. The macrophages were scraped off using a cell scraper (83.3951, Sarstedt, Nümbrecht, Germany) and 50,000 macrophages per well of the 24-well plate were added to the previously compressed PDLFs and incubated at 37 °C for an additional 3 days. Finally, TRAP staining was performed.

### Determination of cell number using the crystal violet assay

The supernatant was removed and adherent cells were washed with 500 μl PBS (14190-094, Gibco™, Carlsbad, CA, USA), then 200 μl crystal violet solution consisting of 2.5 g crystal violet (T123.3, Carl Roth, Karlsruhe, Germany); 0.85 g NaCl (3957, Carl Roth, Karlsruhe, Germany); 20 ml 37% formaldehyde (M4003, Sigma-Aldrich, St. Louis, MO, USA), 150 ml ethanol (32205, Sigma-Aldrich, St. Louis, MO, USA) were added in a total volume of 500 ml per well of a 24-well plate. After 15 min at 37 °C, the plate was washed three times with tap water and lightly tapped out onto a paper towel. The wells were dried at 37 °C for 45 min. After this time, 300 μl of a 33% acetic acid solution (3738.1, Carl Roth, Karlsruhe, Germany) were added per well and measured at a wavelength of 595 nm using an ELISA reader (Multiscan Go, Thermo Fisher Scientific, Waltham, MA, USA).

### Determination of cytotoxicity using the lactate dehydrogenase assay

Lactate dehydrogenase (LDH) release was determined with LDH assay (04744926001; Roche, Basel, Switzerland) according to manufacturer’s instruction. After an incubation period of 30 min in the dark, stop solution was added to each well. Thereafter, measurements were made at wavelengths of 490 and 690 nm (reference) using Multiscan Go (Thermo Fisher Scientific, Waltham, MA, USA).

### RNA isolation and cDNA synthesis

RNA Solv® Reagent (250 µl, R6830-01, VWR international, Radnor, PA, USA) was added directly to the cell culture plates. After a short incubation on ice to destroy the cells, the supernatant was transferred to microreaction tubes followed by the addition of 100 μl chloroform. Samples were mixed for 30 s and then incubated on ice for 15 min. This was followed by centrifugation at 13,000 rpm at 4 °C for 15 min. The clear aqueous phase was transferred to a new tube with 500 μl cold isopropanol (20,842,330; VWR international, Radnor, PA, USA). After inverting, the samples were stored at −80 °C overnight. The following day, samples were centrifuged for 30 min at 4 °C at 13,000 rpm. The pellet was washed twice with 500 μl 80% ethanol (32205, Sigma-Aldrich, St. Louis, MO, USA). After drying, the pellet was solved in 15 μl RNAse-free water (T143, Carl Roth, Karlsruhe, Germany) and concentration of recovered RNA was measured by nano-photometer (Implen, Munich, Germany). To be transcribe RNA into cDNA equal concentrations of RNA were mixed with 2 µl of M‑MLV 5x buffer (M531, Promega, Madison, WI, USA), 0.5 µl of OligodT primer (SO123, Thermo Fisher Scientific, Waltham, MA, USA), 0.5 µl of Random Hexamer primer (SO142, Thermo Fisher Scientific, Waltham, MA, USA), 10 mM dNTPs (L785. 1/2, Carl Roth, Karlsruhe, Germany), 0.5 µl RNAse inhibitor (EO0381, Thermo Fisher Scientific, Waltham, MA, USA) and 0.5 µl reverse transcriptase (M170B, Promega, Madison, WI, USA) in a total volume of 10 µl per sample. The PCR program involved heating the lid to 110 °C, then holding the temperature in the block constant at 37 °C for 1 h, and finally heating to 95 °C for 2 min.

### Quantitative real-time polymerase chain reaction (RT-qPCR)

The aim of the polymerase chain reaction is to exponentially amplify the synthesized cDNA. For this purpose, 1.5 μl cDNA of each sample was mixed with 8.5 μl of the previously prepared primer mix in duplicate into a 96-well plate (712282, Biozym Scientific, Hessisch Oldendorf, Germany), covered with an adhesive optical film (712350, Biozym Scientific, Hessisch Oldendorf, Germany) and briefly centrifuged. The primer mix consisted of 0.25 μl each of forward and reverse primer (Table 1), 3 μl RNAse-free water (T143.5, Carl Roth, Karlsruhe, Germany), and 5 μl Luna Universal qPCR Master Mix (M3003E, New England Biolabs, Ipswich, MA, USA) for each sample. Plates were placed in the thermal cycler (Mastercylcer® Realplex^2^, Eppendorf, Hamburg, Germany) and the appropriate program was started. This involved first heating the plates to 95 °C for 2 min, followed by 45 cycles of 10 s at 95 °C, 20 s at 60 °C, and 8 s at 72 °C. The reference genes *PPIB/RPL22* [13] were used to standardize the target genes. Relative gene expression was determined using the formula 2^−∆∆Cq^ [20].

**Table 1:** Alphabetical list of reference and target gene primers used for real-time quantitative polymerase chain reaction (RT-qPCR) Alphabetische Liste der für die RT-qPCR („real-time quantitative polymerase chain reaction“) verwendeten Referenz- und Zielgen-Primer

Gene	Gene name	5′-forward-primer-3′	5′-reverse-primer-3′
*COL1A2*	Collagen-1-α‑2	AGAAACACGTCTGGCTAGGAG	GCATGAAGGCAAGTTGGGTAG
*IL1B*	Interleukin-1β	ATGACCTGAGCACCTTCTTTCCCT	GCATCGTGCACATAAGCCTCGTTA
*IL6*	Interleukin‑6	TGGCAGAAAACAACCTGAACC	CCTCAAACTCCAAAAGACCAGTG
*PPIB*	Peptidyl-Prolyl-cis-trans-Isomerase B	TTCCATCGTGTAATCAAGGACTTC	GCTCACCGTAGATGCTCTTTC
*RPL22*	Ribosomal 60S-Protein L22	TGATTGCACCCACCCTGTAG	GGTTCCCAGCTTTTCCGTTC

### Western blot analysis

Protein purification was performed with CelLytic™ M (C2978, Sigma-Aldrich, St. Louis, MO, USA) supplemented with proteinase/phosphatase inhibitor (78440, Thermo Fisher Scientific, Waltham, MA, USA). Concentration of proteins was determined using Roti-Quant (K015.3, Carl Roth, Karlsruhe, Germany). Same amounts of proteins were mixed with 6 × sample buffer (3.75 ml Tris/HCl pH 6.8 (T1503, Sigma-Aldrich, St. Louis, MO, USA), 3 ml glycerol (818709, Sigma-Aldrich, St. Louis, MO, USA), 1.2 g 10% SDS (8029.1, Carl Roth, Karlsruhe, Germany), 0.06 g bromophenol blue (108122, Supelco®, St. Louis, MO, USA) ad with water to 10 ml; 0.1 g DTT (dithiothreitol; R0861, Thermo Fisher Scientific, Waltham, MA, USA) per ml) and heated to 70 °C for 7 min. Proteins were separated on 12% polyacrylamide gels at 80 V for the first 30 min, followed by 120 V for 90 min. Separated proteins were transferred to a polyvinylidene difluoride (PVDF) membrane (T830.1, Carl Roth, Karlsruhe, Germany) at 90 V for 90 min. To prevent nonspecific binding of antibodies to the membrane, the membrane was placed at room temperature for at least 1 h in 5% milk (T145.3, Carl Roth, Karlsruhe, Germany) in TBS‑T. This was followed by incubation with the diluted primary antibodies (HSP27 1:3000 (AF1580, R&D Systems, Minneapolis, MN, USA); pHSP27 1:500 (AF2314, R&D Systems, Minneapolis, MN, USA), PTGS2 1:2500 (PA5-1817, Thermo Fisher Scientific, Waltham, MA, USA), RANKL 1:2000 (TA306362, OriGene, Rockville, MD, USA); ACTIN 1:1000 (E1C602, ABIN274248, Antibody-online, Aachen, Germany)). Incubation was performed at 4 °C overnight. The next day, the membrane was washed three times with TBS‑T for 5 min each at room temperature and then incubated with the secondary antibody (1:5000, 611-1302, Rockland Immunochemicals, Pottstown, PA, USA) for 1 h at room temperature. After this time, the membrane was washed again three times with TBS‑T for 5 min at room temperature. Subsequently, the membrane was coated with Luminata Forte Western HRP Substrate (WBLUF0100, Sigma-Aldrich, St. Louis, MO, USA) and signals were detected with the documentation system VWR Genoplex (VWR international, Radnor, PA, USA). Densitometric analysis was performed using ImageJ software (National Institutes of Health, Bethesda, USA). To test for further protein expression, the membrane was incubated three times for 10 min with TBS‑T followed by 20 min in Re-Blot Plus Mild (2502, Merck, Darmstadt, Germany). This was followed by two more washes with TBS‑T for 10 min. After 1 h in 5% milk in TBS‑T, the membrane could be incubated with another antibody overnight at 4 °C.

### Enzyme-linked immunosorbent assay

The commercially available enzyme-linked immunosorbent assay (ELISA) kit TNFRSF11B (EHTNFRSF11B, Thermo Fisher Scientific, Waltham, MA, USA) was used to quantify osteoprotegerin (OPG) at the protein level. Initially, PDLF cell culture supernatants were completely thawed, diluted 1:10 with the corresponding buffer of the ELISA kit and further proceeded according to the manufacturer’s instructions. Photometric measurement was performed using Multiscan Go (Thermo Fisher Scientific, Waltham, MA, USA) at 450 and 550 nm (reference).

### Tartrate resistant acid phosphatase staining

To prepare the TRAP (tartrate resistant acid phosphatase) staining solution, 2 mg of phosphate disodium salt (N-5000, Sigma-Aldrich, St. Louis, MO, USA) was first dissolved in 200 μl of H_2_O_dd_. Acetate buffer was prepared by mixing 35.2 ml 0.2 M sodium acetate solution, 14.8 ml 0.2 M acetic acid solution and 50 ml H_2_O_d_. TRAP buffer pH 5.0 was then freshly prepared with 10 ml of acetate buffer, 2 ml of 0.3 M sodium tartrate, 200 μl naphthol AS-MX phosphate (10 mg/mL; N5000, Sigma-Aldrich, St. Louis, MO, USA), 20 μl Triton X‑100 (T8787, Sigma-Aldrich, St. Louis, MO, USA) and 7.78 ml H_2_O_dd_. TRAP buffer was preheated at 37 °C and then 0.3 mg/ml Fast Red Violet LB Stain (F-3381, Sigma-Aldrich, St. Louis, MO, USA) were dissolved in TRAP buffer. The TRAP staining solution was preheated at 37 °C until use. Cells were washed with prewarmed PBS (14190-094, Gibco™, Carlsbad, CA, USA) and fixed with 10% glutaraldehyde (G-5882, Sigma-Aldrich, St. Louis, MO, USA) at 37 °C for 15 min. After washing the cells twice with PBS, TRAP staining solution was added and incubated for 10 min at 37 °C. Then TRAP staining solution was removed and stained cells were washed with PBS. TRAP-positive cells (red) were counted using an Olympus IX50 microscope (Olympus, Shinjuku, Japan).

### Statistical analysis

Statistical analysis was performed using the program GraphPadPrism 9.2. Prior to statistical analysis, all absolute data values were divided by the respective arithmetic mean of the control group without mechanical loading to obtain normalized data values relative to these controls. Data are presented as single point diagrams with each symbol representing one data point. In addition, the horizontal lines in the graphs show the mean values and the vertical lines the standard error of the mean. Normal distribution of the data was examined with the Shapiro–Wilk test and homogeneity of the groups was determined with the Brown–Forsythe test. Depending on the normal distribution and homogeneity of the data, the following tests were performed: analysis of variance (ANOVA) followed by Holm–Šídák post hoc tests, Welch-corrected ANOVA followed by Dunnett’s T3 post hoc tests, with two groups to be compared: Mann–Whitney U test. Differences were considered statistically significant at *P* < 0.05.

## Results

### HSP27 phosphorylation in PDLF after mechanical strain with different force magnitudes

**Fig. 1:**
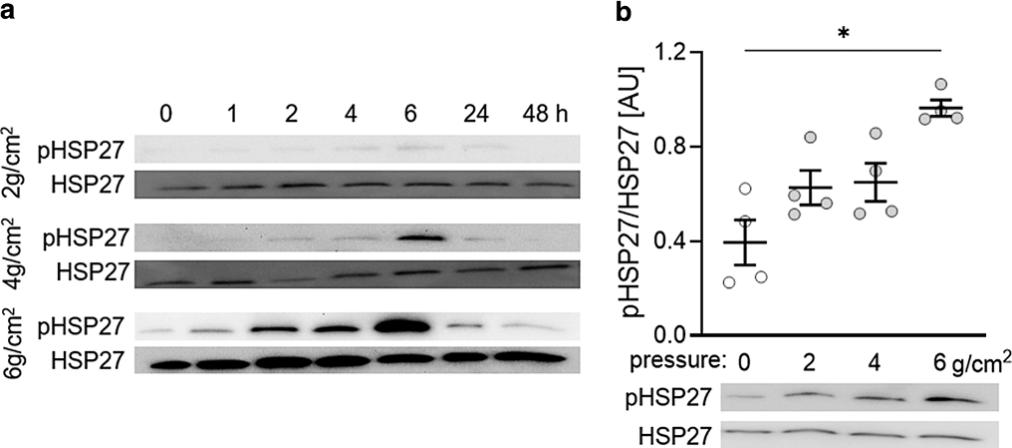
Phosphorylation of heat shock protein 27 (HSP27) after mechanical strain with different force magnitudes for different time spans (**a**). Densitometric analysis of HSP27 phosphorylation in relation to HSP27 expression after exposing human periodontal ligament fibroblasts (PDLF) to different force magnitudes for 6 h (**b**). Statistics: Welch analysis of variance (ANOVA) with Dunnett’s T3 multiple comparisons tests; *P < 0.05 Phosphorylierung von HSP27 (Hitzeschockprotein 27) nach mechanischer Belastung mit unterschiedlichen Kraftstärken für verschiedene Zeitspannen (**a**). Densitometrische Analyse der HSP27-Phosphorylierung im Verhältnis zur HSP27-Expression nach 6‑stündiger Exposition von PDLF (parodontale Ligamentfibroblasten) mit unterschiedlichen Kraftstärken (**b**). Statistik: Welch-ANOVA („analysis of variance“) mit Dunnett-T3 Post-hoc-Test; *p < 0,05

First, we wanted to investigate the optimal timepoints and force magnitudes to study the role of HSP27 phosphorylation on the expression profile of PDLF. With a force size of 2 g/cm^2^, hardly any signal could be obtained for pHSP27 (Fig. 1a). With 4 g/cm^2^, increased HSP27 phosphorylation was evident after 6 h. With 6 g/cm^2^ this was already observed after 2 h. Again, a peak of HSP27 phosphorylation was evident after 6 h (Fig. 1a). The densitometric evaluation of the HPS27 phosphorylation after exposure to different force magnitudes for 6 h showed a significant difference only at 6 g/cm^2^ compared to the untreated controls (*P* = 0.013; Fig. 1b).

### Effects of high force magnitudes on PDLF

**Fig. 2:**
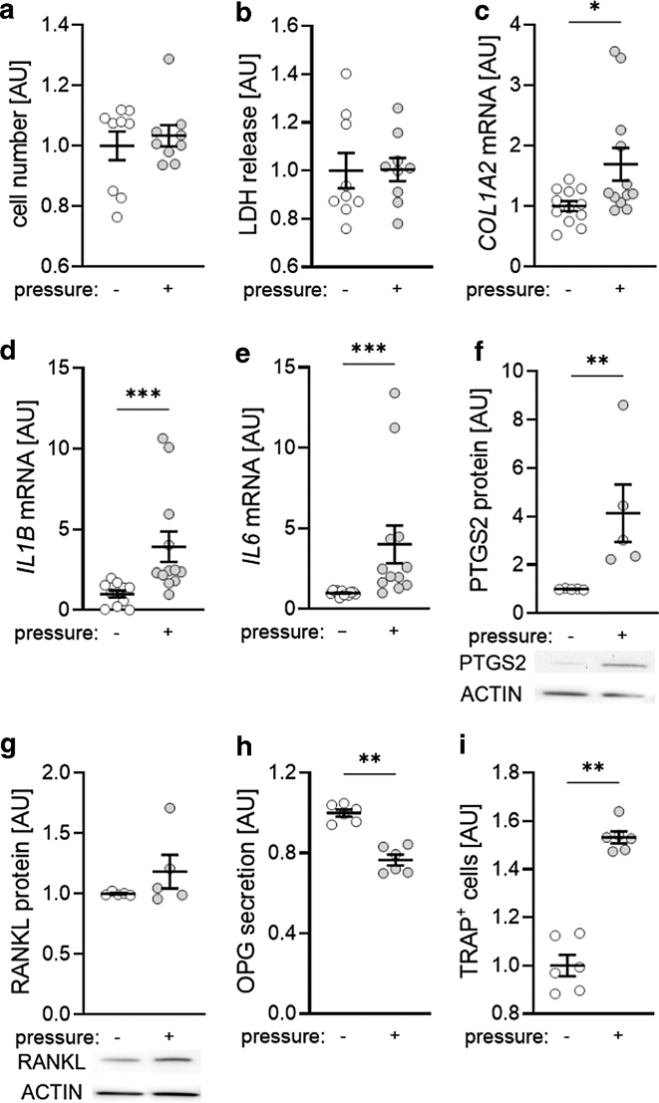
Cell number (**a**) and lactate dehydrogenase (LDH) release (**b**) after exposure of human periodontal ligament fibroblasts (PDLF) to 6 g/cm^2^ for 6 h. Expression of *COL1A2* mRNA (**c**), *IL1B* mRNA (**d**), *IL6* mRNA (**e**), PTGS2 protein (**f**), RANKL protein (**g**) and OPG protein (**h**) as well as determination of TRAP^+^ cells in a coculture model using THP1 macrophages (**i**) after exposure of PDLF to 6 g/cm^2^ for 6 h. Statistics: Mann–Whitney U test; *P < 0.05; **P < 0.01; ***P < 0.001 Zellzahl (**a**) und LDH(Laktatdehydrogenase)-Freisetzung (**b**) nach 6‑stündiger Exposition von PDLF (parodontale Ligamentfibroblasten) mit 6 g/cm^2^. Expression von *COL1A2* mRNA (**c**), *IL1B* mRNA (**d**), *IL6* mRNA (**e**), PTGS2-Protein (**f**), RANKL-Protein (**g**) und OPG-Protein (**h**) sowie Bestimmung von TRAP^+^-Zellen in einem Kokulturmodell mit THP1-Makrophagen (**i**) nach 6‑stündiger Exposition von PDLF mit 6 g/cm^2^. Statistik: Mann-Whitney-U-Test; *p < 0,05; **p < 0,01; ***p < 0,001

Most experiments dealing with PDLF and compressive force were conducted with 2 g/cm^2^ for up to 48 h. After identifying a force magnitude of 6 g/cm^2^ for up to 6 h as optimal for HSP27 phosphorylation, we first wanted to determine any possible cytotoxic effects. Neither the cell number (*P* = 0.546; Fig. 2a) nor LDH release (*P* = 0.666; Fig. 2b) were affected by the used conditions. Gene expression of Collagen-1-α‑2 (*COL1A2*) was significantly increased after compressive force treatment (*P* = 0.039; Fig. 2c) indicating increased collagen synthesis. Expression of inflammatory genes like Interleukin-1β (*IL1B*; *P* < 0.001; Fig. 2d) and *IL6* (*P* < 0.001; Fig. 2e) was upregulated within 6 h of compression with 6 g/cm^2^. Protein expression of prostaglandin endoperoxide synthase‑2 (PTGS2) was also upregulated (*P* < 0.001; Fig. 2f). We detected no effect on receptor activator of NF-κB ligand (RANKL) protein expression (*P* = 0.744; Fig. 2g), while osteoprotegerin (OPG) protein secretion was significantly downregulated after 6 h compression with 6 g/cm^2^ (*P* < 0.001; Fig. 2h) resulting in more TRAP^+^ cells in a coculture model using THP1 macrophages (*P* = 0.002; Fig. 2i). The examined force magnitude and application time is therefore suitable to study effects on inflammatory and bone remodeling mediators.

### Effects of phosphorylation of HPS27 on cell vitality of PDLF

**Fig. 3:**
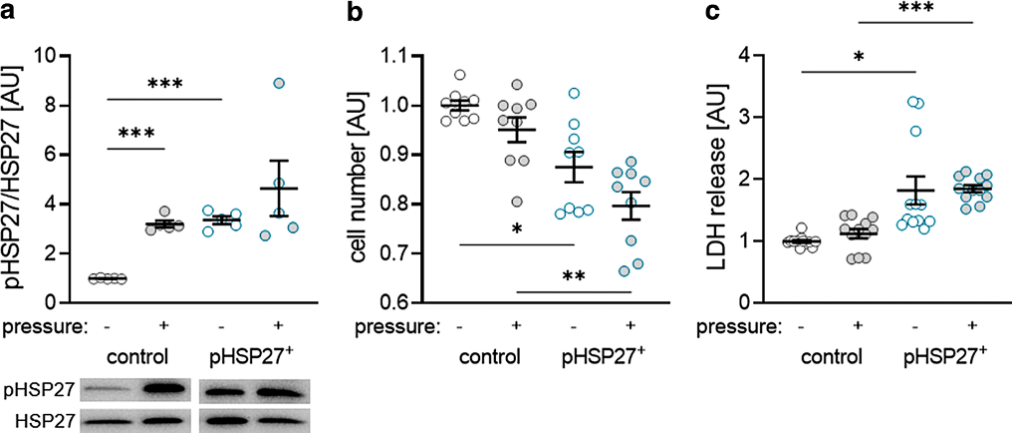
Heat shock protein 27 (HSP27) phosphorylation was upregulated after compression and transfection of pHSP27^+^ (**a**). Transfection of pHSP27^+^ reduced the cell number (**b**) and increased lactate dehydrogenase (LDH) release (**c**) in periodontal ligament fibroblasts (PDLF) without and with compression. Statistics: Welch analysis of variance (ANOVA) with Dunnett’s T3 multiple comparisons tests; *P < 0.05; **P < 0.01; ***P < 0.001 Die HSP27(Hitzeschockprotein 27)-Phosphorylierung wurde nach Kompression und Transfektion von pHSP27+ hochreguliert (**a**). Die Transfektion von pHSP27+ reduzierte die Zellzahl (**b**) und erhöhte die LDH(Laktatdehydrogenase)-Freisetzung (**c**) in PDLF (parodontale Ligamentfibroblasten) ohne und mit Kompression. Statistik: Welch-ANOVA („analysis of variance“) mit Dunnett-T3 Post-hoc-Test; *p < 0,05; **p < 0,01; ***p < 0,001

To investigate the influence of phosphorylation of HSP27 on the sterile inflammatory response occurring during orthodontic tooth movement in the periodontal ligament, PDLF were transfected with pHSP27^+^, which contains the human HSP27 gene with three times aspartate instead of serine, corresponding to permanent phosphorylation. Control cells were transfected with an empty vector. Accordingly, we detected increased HSP27 phosphorylation after compression and transfection with the pHSP27^+^ (*P* < 0.001; Fig. 3a). Compression failed to further increase pHSP27 (*P* = 0.716; Fig. 3a). Surprisingly, transfection with pHSP27^+^ resulted in reduced cell numbers without (*P* = 0.011) and with compression (*P* = 0.003; Fig. 3b). In line with that, we detected increased LHD release after transfection (without pressure: *P* = 0.016; pressure: *P* < 0.001; Fig. 3c) indicating a cytotoxic effect of the plasmid pHSP27^+^.

### Effects of phosphorylation of HPS27 on the expression of collagen and inflammatory mediators in PDLF

**Fig. 4:**
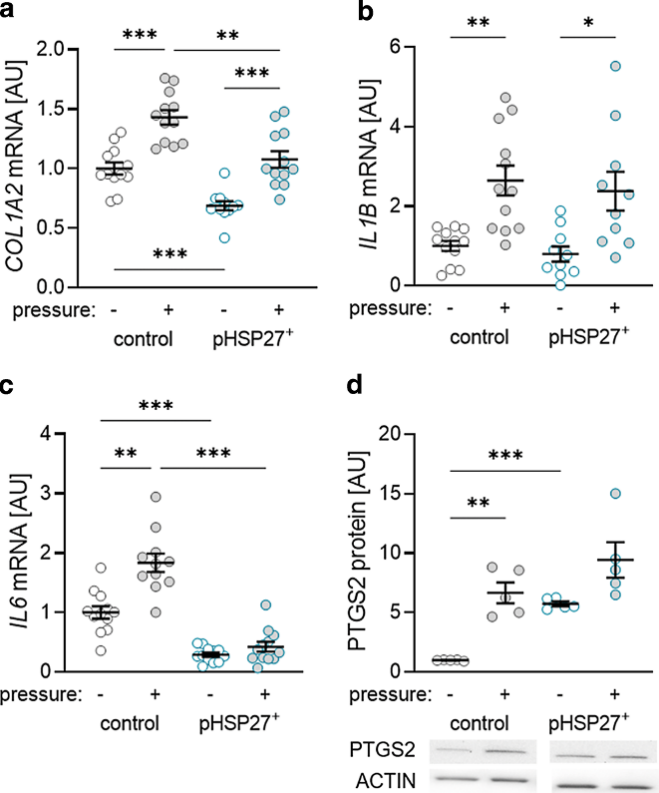
Gene expression of *COL1A2* (**a**), *IL1B* (**b**) and *IL6* (**c**) as well as PTGS2 protein expression (**d**) in periodontal ligament fibroblasts (PDLF) without and with compression. Statistics: Welch analysis of variance (ANOVA) with Dunnett’s T3 multiple comparisons tests; *P < 0.05; **P < 0.01; ***P < 0.001 Genexpression von *COL1A2* (**a**), *IL1B* (**b**) und *IL6* (**c**) sowie PTGS2-Proteinexpression (**d**) in PDLF (parodontale Ligamentfibroblasten) ohne und mit Kompression. Statistik: Welch-ANOVA („analysis of variance“) mit Dunnett-T3 Post-hoc-Test; *p < 0,05; **p < 0,01; ***p < 0,001

Extracellular matrix remodeling occurs during orthodontic treatment. Accordingly, we detected increased *COL1A2* gene expression after transfection with the control and pHSP27^+^ plasmid after mechanical strain (*P* < 0.001; Fig. 4a). However, pHSP27^+^ transfection reduced *COL1A2* gene expression without (*P* < 0.001) and with pressure application (*P* = 0.003) indicating a regulatory effect of HSP27 phosphorylation on *COL1A2* gene expression.

As the sterile inflammatory process is crucial for orthodontic tooth movement, we investigated the effects of phosphorylation of HSP27 on the expression of inflammatory genes and proteins. Mechanical strain increased *IL1B* gene expression after transfection with an empty vector (control; *P* = 0.004) and with pHSP27^+^ (*P* = 0.039; Fig. 4b). Transfection with the permanently phosphorylated HSP27 plasmid had no effect on *IL1B* gene expression without (*P* = 0.836) and with mechanical strain (*P* = 0.986; Fig. 4b). As expected, *IL6* mRNA levels were elevated after compression in cells transfected with the empty vector (*P* = 0.001; Fig. 4c). Transfection with pHSP27^+^ reduced IL6 gene expression without and with mechanical strain (*P* < 0.001; Fig. 4c). PTGS2 protein expression was increased with mechanical strain after transfection with the empty vector (*P* = 0.009; Fig. 4d). Transfection with pHSP27^+^ led to an increase of PTGS2 protein expression without pressure application (*P* < 0.001; Fig. 4d). There was no significant difference due to pHSP27^+^ transfection after mechanical strain (*P* = 0.448).

### Effects of constitutive phosphorylation of HSP27 on bone remodeling mediated by PDLF

**Fig. 5:**
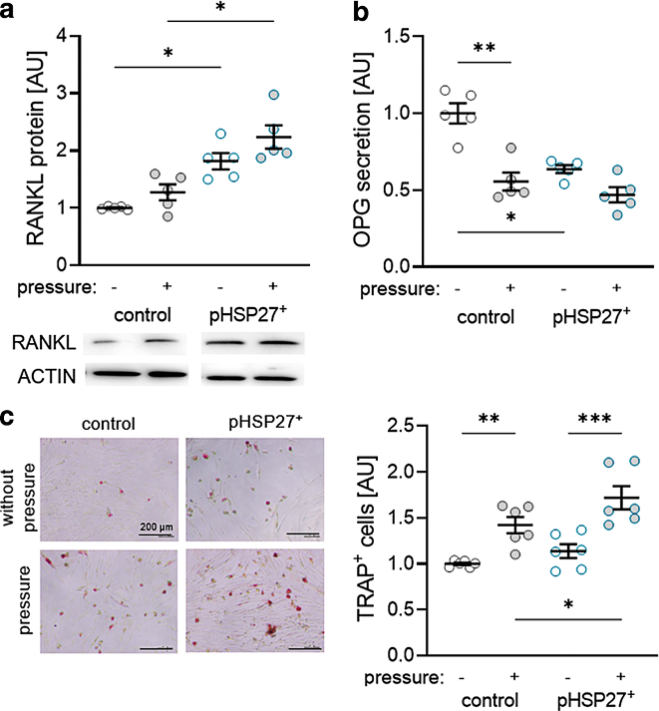
Receptor activator of NF-κB ligand (RANKL) protein expression (**a**) and osteoprotegerin (OPG) secretion (**b**) in periodontal ligament fibroblasts (PDLF) without and with compression as well as assessment of TRAP^+^ cells (**c**) in a coculture model using THP1 macrophages. Statistics: Welch analysis of variance (ANOVA) with Dunnett’s T3 multiple comparisons tests; *P < 0.05; **P < 0.01; ***P < 0.001 RANKL(„receptor activator of NF-κB ligand“)-Proteinexpression (**a**) und OPG(Osteoprotegerin)-Sekretion (**b**) in PDLF (parodontale Ligamentfibroblasten) ohne und mit Kompression sowie Bestimmung der TRAP+ Zellen (**c**) in einem Kokulturmodell mit THP1-Makrophagen. Statistik: Welch-ANOVA („analysis of variance“) mit Dunnett-T3 Post-hoc-Test; *p < 0,05; **p < 0,01; ***p < 0,001

Orthodontic tooth movement depends on bone resorption facilitated by osteoclasts. Osteoclastogenesis itself is crucially regulated by RANKL and its decoy receptor OPG. With the setup of 6 g/cm^2^ for 6 h, we detected no increased RANKL protein expression after transfection with an empty control vector and mechanical loading (*P* = 0.341; Fig. 5a). Transfection with pHSP27^+^ increased RANKL expression under control (*P* = 0.015) and pressure conditions (*P* = 0.021; Fig. 5a). Compressive force of 6 g/cm^2^ for 6 h decreased OPG secretion, when cells were transfected with the control plasmid (*P* = 0.004; Fig. 5b). Treatment with pHSP27^+^ significantly decreased OPG secretion without mechanical loading (*P* = 0.013; Fig. 5b). Accordingly, we detected more TRAP^+^ cells after pressure application and pHSP27 transfection (*P* = 0.048; Fig. 5c).

## Discussion

Muraoka et al. reported increased HSP27 expression and phosphorylation in the periodontal ligament of mice after force application [23]. This effect was already observed 10 min after the load application and reached a peak after nine hours. This was comparable to our results as we observed a maximum of HSP27 phosphorylation after six hours. In contrast to the results of Muraoka et al., we observed no changes in HSP27 expression. Phosphorylation of HSP27 affected collagen, IL6 and PTGS2 expression as well as the RANKL/OPG ratio resulting in the induction of an increased number of TRAP^+^ cells after compressive force treatment.

Remodeling of the extracellular matrix is crucial for orthodontic tooth movement. We observed decreased *COL1A2* mRNA levels after transfection with pHSP27^+^. Hirano et al. and Deng et al. reported that HSP27 overexpression increased collagen synthesis in fibroblasts [9] or in alveolar type II epithelial cells [6]. Also, HSP70 and HSP47 were associated with collagen secretion in PDLF during mechanical loading [24].

IL6 is the classic proinflammatory cytokine associated with bone resorption [43]. Constitutively phosphorylated HSP27 led to a decrease in *IL6* gene expression by PDLF. This observation is consistent with the results of Zhang et al. in intestinal epithelial cells [49]. Sur et al. also identified an involvement of HSP27 in the NF-κB signaling pathway in keratinocytes. They described an increased production of inflammatory mediators as a consequence downregulation of HSP27 [36]. Accordingly, a decrease in *IL6* expression could be due to increased levels of phosphorylated HSP27. The increased RANKL/OPG ratio seems to be in apparent contradiction to the lowered expression of *IL6*. Marciniak et al. (2019) investigated the role of heat shock protein 70 (HSP70) during mechanical loading by inhibiting HSP70 [21]. They used a compressive force of 2 g/cm^2^ and observed increased *IL6* and *IL8* gene expression after compression. Inhibition of HSP70 and simultaneous pressure application resulted in an even higher gene expression of *IL6* and *IL8*, suggesting a protective function of HSP70 [21]. Wolf et al. investigated the effect of heat pretreatment on PDLF [41]. PDLF were subjected to an elevated temperature of 43 °C before force application. This treatment significantly reduced protein secretion of IL6 and IL8. Therefore, heat shock proteins are considered a promising therapeutic option to minimize undesirable side effects during orthodontic treatments [41]. For the experiments, PDLF were transfected with HSP27^+^, which is characterized by having aspartate instead of the three serine residues. Aspartate, like the phosphate ion, carries a negative charge. Thus, this plasmid corresponds to permanent phosphorylation and acquires phospho-mimetic properties [35]. In its native form, HSP27 is also present in a dephosphorylated state and can only form large oligomers and perform its chaperone function in this way [15]. Phosphorylation of HSP27 causes oligomers to dissociate [34]. These results may imply that there is some protective function against the generation of proinflammatory factors when HSP27 is not phosphorylated and thus is in the form of oligomers. This allows HSP27 to perform its chaperone function.

Compression with 6 g/cm^2^ for 6 h led to an increase in the RANKL/OPG ratio, which was due to a reduction in OPG secretion and increased PTSG2 protein expression. PTGS2 is critically involved in prostaglandin synthesis [7, 40]. PGE2 has been shown to promote RANKL production and inhibit OPG expression in osteoblasts [18]. Since inflammatory mediators promote RANKL production and inhibit OPG synthesis, one would expect a reduction in this ratio. However, as described at the beginning, RANKL and OPG synthesis depend on numerous cellular factors in addition to inflammatory mediators. These include the cytoskeleton [25], integrins [30, 48], connexins [16, 44], and ion channels [17, 32]. Thus, it could be that HSP27 interferes with another, as yet unknown, signaling pathway, thereby influencing the RANKL/OPG ratio in favor of RANKL. However, further research is needed to test this conjecture. Overall, HSP27 could be a promising target in orthodontic treatments.

## Conclusion

Phosphorylation of HSP27 could accelerate orthodontic tooth movement by increasing the RANKL/OPG ratio, which leads to increased differentiation of osteoclasts and thus more bone resorption. In addition, painful inflammatory responses could possibly be attenuated.

## Supplementary Information

Supplemental Fig. 1: (a) Schematic presentation of the different zirconium oxide plates for pressure application. (b) Timeline to the transfection experiments. (c) Timeline to the coculture experiments
