# Supplementary material for: Impact of phosphorylation of heat shock protein 27 on the expression profile of periodontal ligament fibroblasts during mechanical strain
Source: J Orofac Orthop. 2022 Apr 21;84(Suppl 2):143–53. doi: 10.1007/s00056-022-00391-w (PMC10126016; doi:10.1007/s00056-022-00391-w)
Supplement: Supplementary file 1 — Supplemental Fig. 1: (a) Schematic presentation of the different zirconium oxide plates for pressure application. (b) Timeline to the transfection experiments. (c) Timeline to the coculture experiments [file 56_2022_391_MOESM1_ESM.pdf]

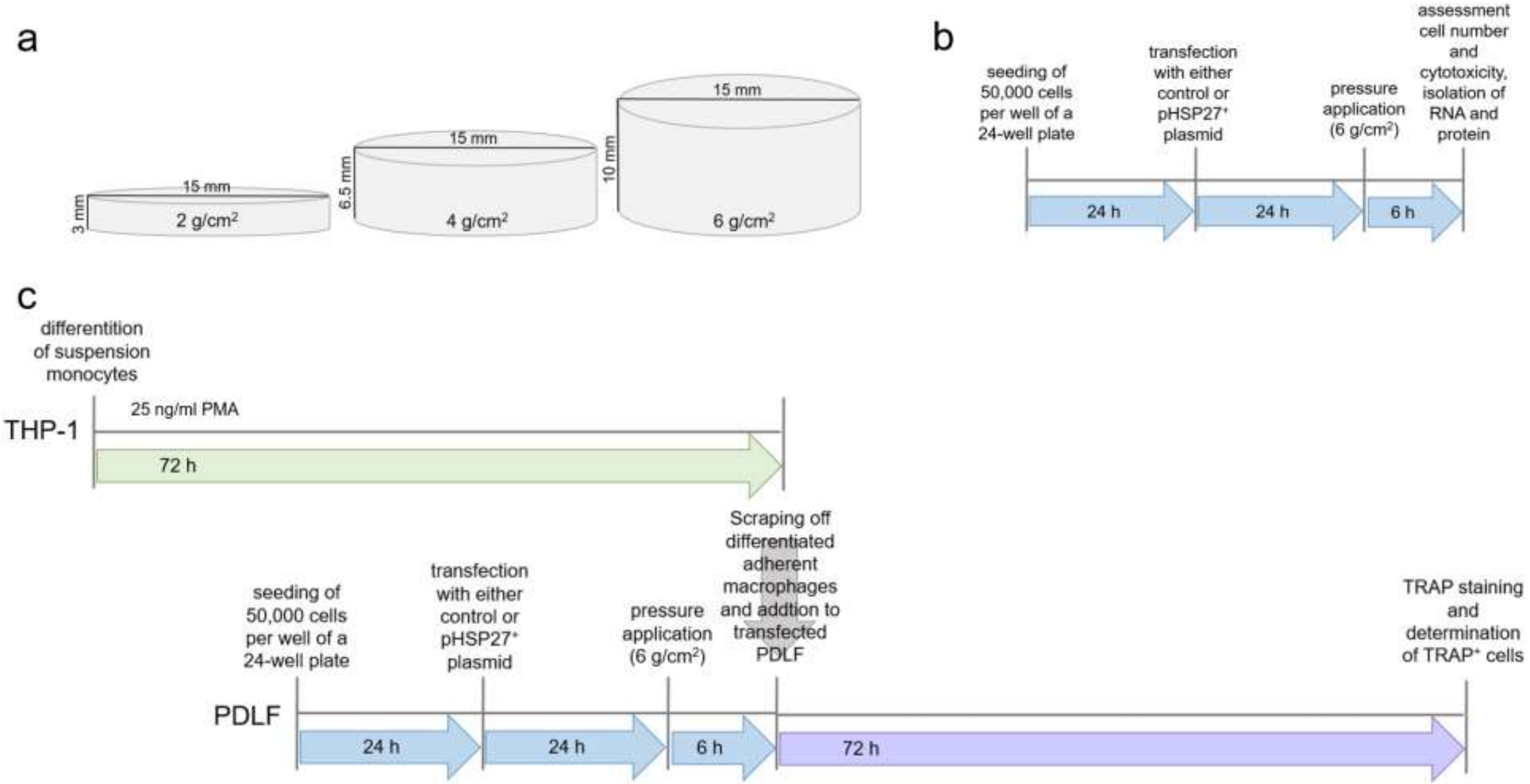

Supplemental Figure 1: (a) Schematic presentation of the different zirconium oxide plates for pressure application. (b) Timeline to the transfection experiments. (c) Timeline to the coculture experiments.
